# Secretases: potential and roadblocks on the way to therapy

**DOI:** 10.1186/1750-1326-8-S1-O22

**Published:** 2013-09-13

**Authors:** Bart De Strooper

**Affiliations:** 1 VIB Center for the biology of Disease, VIB-KULeuven, Belgium

## 

Over the last decade important progress has been made towards the understanding of the molecular pathobiology of Alzheimer’s Disease. One of the major breakthroughs was the identification of presenilin and its crucial role in the gamma-Secretase processing of APP and Notch. Ever since, it has been a major aim to block gamma-Secretase in a safe way. Recent results of trials, in particular the semagacestat trial of Ely-Lilly, indicate that blocking gamma-Secretase in Alzheimer patients can cause important side effects. Significantly, the cognitive condition of patients under semagacestat treatment worsened, suggesting an important role of one of the gamma-Secretase substrates in cognition. The outcome of this trial raises the question whether it will be possible to identify a therapeutic window for gamma-Secretase inhibition in AD. We will show in the current presentation that our knowledge on gamma-Secretase function and regulation has dramatically progressed in the last years. We will explain how different gamma-Secretase complexes have different biological roles, and that selectively targeting them could provide safer drugs. Also increasing insights into structure and function could lead to safer drugs, such as gamma-secretase modulators, but also drugs that specifically interfere with docking of specific substrates to the different gamma-Secretases. Finally insight into the regulation of the gamma-Secretase complex only starts to emerge, which also could open new opportunities for safer drugs.

